# High Genotypic Diversity, Putative New Types and Intra-Genotype Variants of Bovine Papillomavirus in Northeast Brazil

**DOI:** 10.3390/pathogens9090748

**Published:** 2020-09-15

**Authors:** Rebeca P. Figueirêdo, Gabriela F. Santos, Luana B. Oliveira, Lucas A. B. O. Santos, Débora M. Barreto, Alexandre L. Cândido, Ana C. Campos, Edisio O. Azevedo, Marcus V. A. Batista

**Affiliations:** 1 Laboratory of Molecular Genetics and Biotechnology (GMBio), Department of Biology, Center for Biological and Health Sciences, Federal University of Sergipe, São Cristóvão 49100-000, Brazil; rebecafigueiredo@globo.com (R.P.F.); gabrielafsantos@outlook.com.br (G.F.S.); luanabo33@gmail.com (L.B.O.); luccalexandre@gmail.com (L.A.B.O.S.); 2 Centro Universitário Maurício de Nassau, Aracaju 49075-470, Brazil; deborinha.barreto@hotmail.com; 3 Department of Morphology, Center for Biological and Health Sciences, Federal University of Sergipe, São Cristóvão 49100-000, Brazil; virologiacomparada@hotmail.com; 4 Núcleo de Graduação em Medicina Veterinária, Campus do Sertão, Federal University of Sergipe, Nossa Sra. da Glória 49680-000, Brazil; anabutron@gmail.com; 5 Department of Veterinary Medicine, Federal University of Sergipe, São Cristóvão 49100-000, Brazil; eoazevedo@bol.com.br

**Keywords:** bovine papillomavirus, BPV infection, genetic diversity, genetic variation, nonsynonymous mutation, L1 protein

## Abstract

Bovine papillomavirus (BPV) can cause damage to the epithelial and mucosal tissue and currently presents 28 known types. Not all BPV types are associated with the development of cancer in cattle. Studies have shown that variants of human papillomavirus types can present different pathogenic profiles. However, despite the similarity, it is not yet known whether variants of BPV types can also present varying degrees of pathogenicity. Thus, the aim of this study was to evaluate the genetic variability of BPV types and variants isolated in Northeastern Brazil. Samples were obtained from animals with papillomatous lesions. BPV DNA was detected by the amplification of the L1 gene and genotyping was performed by sequencing. Mutations were analyzed in a phylogenetic, structural and functional context. In total, 52 positive samples were obtained and 11 different BPV types were identified in the samples. Ten putative new BPV types were also identified. In addition, several non-synonymous mutations were identified and predicted to alter protein stability, having an impact on immune evasion. The study demonstrated a high genetic diversity of BPV in the region with a large number of mutations identified, serving as a basis for more efficient control measures to be adopted for bovine papillomatosis.

## 1. Introduction

Bovine papillomavirus (BPV) is an oncogenic virus belonging to the *Papillomaviridae* family, which infects the epithelium and mucosa of many animals including cattle. Although papillomaviruses are usually species-specific, BPV has been observed in other animals such as giraffes, buffalo, sheep and horses [1,2,3,4,5,6,7]. BPVs are classified into five genera and 28 types have been identified so far. The genera are: *Deltapapillomavirus*, encompassing BPV1, BPV2, BPV13 and BPV14, *Dyokappapapillomavirus* with types BPV16, BPV18 and BPV22, *Dyoxipapillomavirus* with only BPV7, *Epsilonpapillomavirus* with BPV5, BPV8 and BPV25 and *Xipapillomavirus* with types BPV3, BPV4, BPV6, BPV9, BPV10, BPV11, BPV12, BPV15, BPV17, BPV20, BPV23, BPV24, BPV26 and BPV28. BPV19, BPV21 and BPV27 have not yet been classified [8,9,10].

The *Deltapapillomavirus* genus is considered to be of high risk because it contains BPV types strongly linked to the development of cancer. BPV1 and BPV2 are frequently reported in urothelial tumors and BPV4 in the upper digestive tract. In addition, BPV2, BPV13 and BPV14 are associated with cancer in the urinary bladder of animals that usually feed on bracken fern [9,11,12,13,14,15].

Intra-genotype papillomavirus variants must have more than 98% and less than 100% identity with the L1 gene reference sequence of the closest type [16]. In human papillomavirus (HPV) for example, high-risk types, which infect the mucosa, are associated with persistent infection [17]. Recent studies have reported that different HPV intra-genotype variants, lineages or sub-lineages can be associated with lesions of different risks (low risk, high risk and even cancer) [18,19,20,21,22]. However, there is no report on the specific pathogenic characteristics for BPV intra-genotype variants, but the similarities between HPV and BPV make it possible to question the possibility of genetic variants of BPV also being associated with different pathogenic potentials [9,13,23,24,25,26,27,28,29,30,31].

Given the possibility that different BPV types may have different clinical manifestations of the disease, it is important to know the viral types circulating in a region so that it serves as a basis for a better understanding of the genetic diversity of BPV and more efficient control measures can be adopted for bovine papillomatosis. Thus, the aim of this study was to evaluate genetic diversity based on types and variants of BPV in Northeastern Brazil. Molecular and bioinformatics analyses were used to detect and evaluate the genetic variability of BPV, demonstrating that the region has significant numbers of viral types and mutations with a structural and functional importance for the L1 protein.

## 2. Results

**Figure 1:**
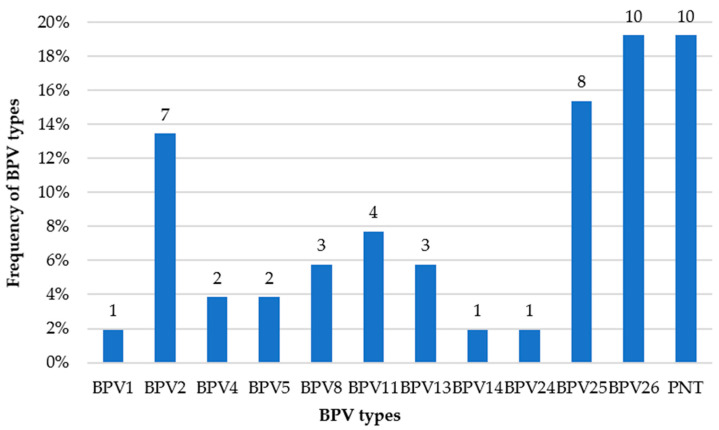
Distribution of the frequency of the bovine papillomavirus (BPV) types identified in the samples. On the Y axis is the percentage in relation to the total number of viruses found, while the X axis identifies which virus is represented by each column. PNT = Putative New Type.

In total, 61 samples were obtained, of which 57 were positive for BPV based on PCR and electrophoresis. After sequencing, 52 sequences showed enough quality for the analyses. Using BLAST, it was possible to identify 11 different BPV types in 42 samples being identified as BPV1, BPV2, BPV4, BPV5, BPV8, BPV11, BPV13, BPV14, BPV24, BPV25 and BPV26. In addition, 10 samples were identified as putative new BPV types; that is, L1 gene sequences that showed less than 90% identity when compared with the reference BPV types (Figure 1).

Several papillomatous lesions were collected per animal allowing the presence of more than one BPV type to be assessed in the same animal. Thus, the presence of co-infections between BPV25 and BPV8, BPV25 and a putative new type (PNT) with a higher identity with BPV3 was observed, as well as with BPV24 and PNT isolates of the genus *Xipapillomavirus* closer to BPV12 and BPV24. Some individuals had infection with different types of the same genus such as BPV25 and BPV8 and other animals were infected with types of BPV belonging to different genera (Table S1).

Approximately 20% of the lesions obtained were infected with BPV types that were still not characterized. The highest percentage of identity with a reference type among the PNTs was 87.8%, while the lowest was 74.1%. These isolates, identified as PNTs of BPV, showed a higher identity with the reference types BPV3, BPV12, BPV24 and BPV26 (Table 1). It was observed that a greater similarity of the samples obtained in this study with non-reference sequences deposited in GenBank identified in the Brazilian states of Acre, Paraná and Santa Catarina, in addition to a sequence from China (KF751802). Despite having a greater identity with these sequences, four PNTs identified in this study did not belong to the same type of these isolate since they had less than 90% identity among them (Table 1).

Among the 42 samples with identified BPV types, 30 were identified as variants and eight as subtypes, as can be seen in Table S1. Four BPV26 subtypes were identified that presented up to 9% nucleotide difference when compared with the reference sequence (Table S2).

**Figure 2:**
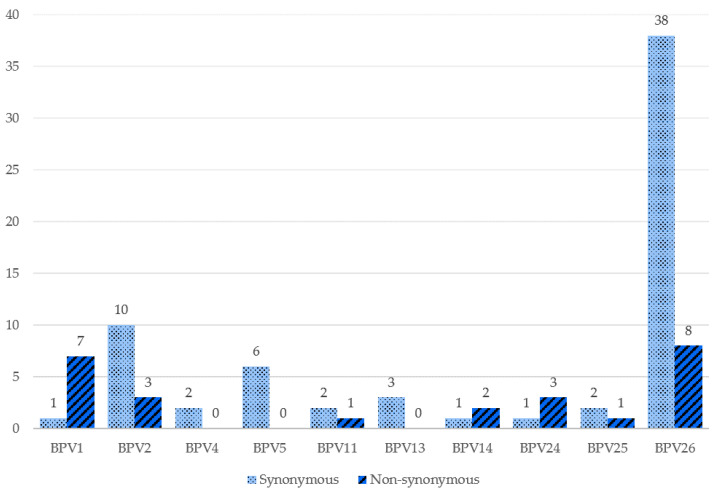
Variable sites by BPV type. Each pair of bars represents the number of mutations found in each type of BPV, the lightest and dotted being the synonymous mutations and the darkest and striped being the non-synonymous mutations found for that BPV type.

For the evaluation of mutations for the L1 gene, the sequences of the BPV samples were compared with the reference sequences of the respective viral type. Figure 2 shows the distribution of the number of variable sites by BPV type. Of the 11 BPV types found, seven types of BPV presented samples with non-synonymous mutations. Regarding the total number of samples, 25 showed non-synonymous mutations when comparing the L1 gene sequences of the isolates with the reference sequences.

Eight mutations were observed in the BPV1 L1 gene sequence, of which seven were non-synonymous (Table S3). Two mutations were found in the BPV4 L1 sequences and all of them were synonymous (Table S4). BPV5 also presented only synonymous mutations with six mutations in total (Table S5). BPV11 isolates presented two synonymous and one non-synonymous mutations (Table S6). Three synonymous mutations were found in the BPV13 sequences (Table S7). BPV14 presented two non-synonymous and one synonymous mutations (Table S8). BPV24 presented three non-synonymous and one synonymous mutations (Table S9). Two synonymous and one non-synonymous mutations were found in BPV25 (Table S10). BPV2 and BPV26 showed the highest number of mutations; BPV2 with 10 synonymous and three non-synonymous mutations (Table S11) and BPV26 with 38 synonymous and eight non-synonymous mutations (Table S12). It is important to highlight that some of these mutations took place in the same codon, affecting the same amino acid.

**Figure 3:**
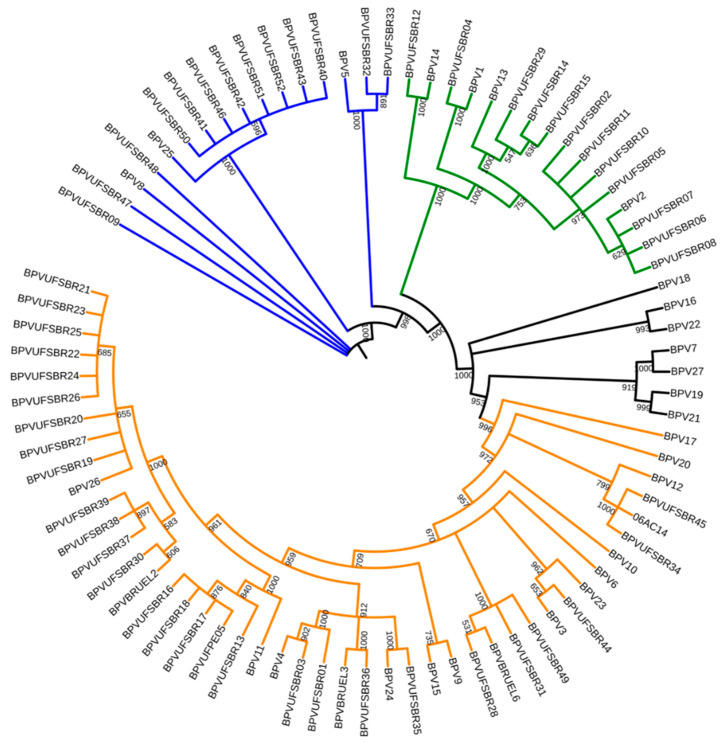
Maximum likelihood phylogenetic tree containing the sequences obtained in this study, the reference sequences and the isolates that showed a higher sequence identity with the putative new types of BPV. Bootstrap values are represented in absolute numbers based on a scale from 0 to 1000 replicates. Only bootstrap values greater than 500 are shown. In orange, the clades with BPV types of the *Xipapillomavirus* genus, in green *Deltapapillomavirus* and in blue *Epsilonpapillomavirus*.

BPV types belonging to three different genera were found: *Deltapapillomavirus*, *Xipapillomavirus* and *Epsilonpapillomavirus*. At least one sample of each type of BPV belonging to the *Deltapapillomavirus* genus was found, totaling 12 isolates from this genus. The *Epsilonpapillomavirus* genus had the second highest number of representatives found with a total of 13 isolates among the BPV types 5, 8 and 25. The *Xipapillomavirus* genus had the highest occurrence, with 27 isolates. In this genus, BPV4, BPV11, BPV24 and BPV26 were found in addition to the 10 isolates of putative new types of BPV (Figure 3). In general, the phylogenetic tree presented well-supported branches with clusters representing the different genera of BPV. As expected, the BPV26 subtypes grouped more distantly from the branch that contained the reference sequence and the variants. The same can be seen in the branches related to BPV2. PNTs were grouped close to their respective reference types but they were more closely grouped with the non-reference isolates obtained from Genbank, which showed greater identity with them (Figure 3).

The BPV types that presented non-synonymous mutations had their proteins modeled so that it was possible to analyze the effect of these mutations on the structure of the BPV L1 protein. All 3D models of BPV L1 proteins showed more than 90% of the amino acid residues in the allowed regions in the Ramachandran plot, the lowest being 90.3% and the highest 91.4%, which evidences the quality and viability of the predicted models. For BPV1, the crystal structure of the L1 protein obtained from the Protein Data Bank was used. For the modeling of the L1 protein of BPV types 2, 14 and 25, the crystal structure of BPV1 was used as a template and to model the protein of BPV types 11, 24 and 26, the crystal structure of L1 of HPV16 was used as a template for presenting a higher identity.

**Figure 4:**
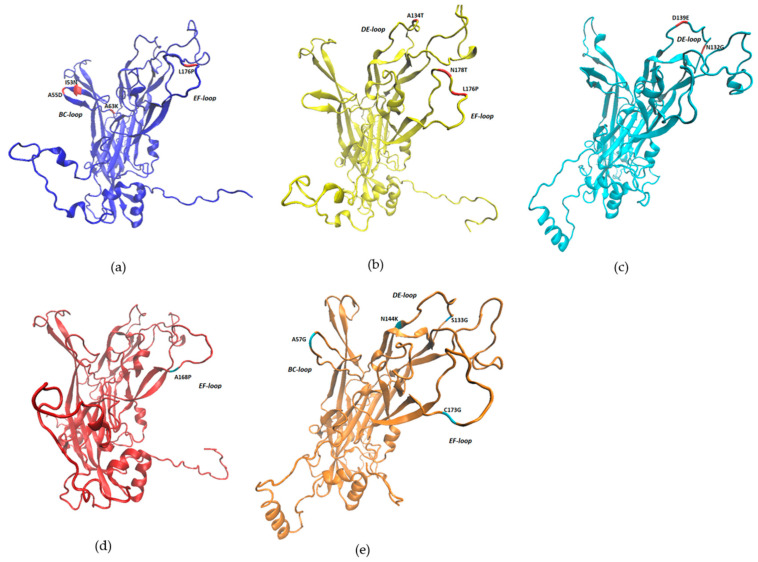
3D structures of BPV L1 protein with the location of the non-synonymous mutations identified in the hypervariable surface loops: (**a**) BPV1 crystal structure obtained from the Protein Data Bank (code PDB: 3IYJ) showing the mutations in the BC and EF loops; (**b**) Model of the BPV2 3D structure with the mutations in the DE and EF loops; (**c**) Model of the 3D structure of BPV24 with the mutations in the DE loop; (**d**) BPV25 3D structure model with the EF loop mutation; (**e**) 3D structure model of BPV26 with mutations in loops BC, DE and EF.

In the structures of the BPV L1 protein, the hypervariable surface loop regions BC, DE, EF, FG and HI were mapped, which have already been identified in HPV as regions of neutralizing epitopes [32,33]. In total, 14 non-synonymous mutations identified in this study were found in these loop regions, one of which (L176P) was identified in both BPV1 and BPV2. In BPV1, I53N, A55D and A63K mutations were identified in the BC loop region and L176P in the EF loop. In BPV2, the A134T mutation was identified in the DE loop and L176P and N178T mutations in the EF loop. In BPV24, mutations N132G and D139E were identified in the DE loop region. In BPV25, the A168P mutation was identified in the EF loop. In BPV26, the mutation A57G was found in the BC loop, the S133G and N144K mutations in the DE loop and C173G in the EF loop (Figure 4).

The samples that showed non-synonymous mutations were also analyzed with an emphasis on the effect of each mutation on the stability of the structure of the BPV L1 protein (Table 2). For the mutations found in the regions of the neutralizing epitopes, the analyses showed a reduction in structural stability for I53N, A55D and A63K in BPV1, A134T and L176P in BPV2, A168P in BPV25 and C173G in BPV26. The L176P mutations of BPV1, N178T of BPV2, N132G and D139E of BPV24, as well as A57G, S133G and N144K of BPV26, which are found in the hypervariable surface loop regions, showed increased stability (Table 2).

## 3. Discussion

The study demonstrated a high genotype diversity of BPV in a region of the Northeast of Brazil as well as a great intra-genotypic genetic variability with the identification of various BPV variants and subtypes. This is evidenced by the identification of 11 different types of BPV, eight subtypes and 30 intra-genotype variants in addition to 10 samples identified as putative new types of BPV. Thus, these results are relevant because they show that, as observed in HPV, there is also a great genetic diversity in BPV with a high number of types and variants that have yet to be discovered.

The high occurrence of BPV2 has been reported in previous studies [34,35,36,37]; however, BPV25 and BPV26 are not frequently reported. The low frequency of BPV1 has also been reported in studies carried out in the Northeast region of Brazil [34,37]. As they are recently discovered types, there are still no studies on the prevalence of BPV25 and BPV26. However, the high frequency found of BPV25 and BPV26 in the region highlights the possibility that they are BPV types that may have a high incidence in Brazil. The high number of mutations, variants and subtypes observed in BPV26 is also an important data to be observed especially as it is a newly characterized virus but which has a high intra-genotypic diversity. This finding presents new research opportunities in the region, focusing on the evaluation of the clinical, epidemiological and pathological aspects of this type of BPV.

In addition to the high genetic diversity, the presence of BPV1, BPV2, BPV4, BPV13 and BPV14 were observed, which are associated with the development of cancer in the animals. BPV1 [9,38], BPV2 [4,9,38], BPV13 [13,38] and BPV 14 [14,38] are related to the appearance of bladder cancer while BPV4 is linked to cancer in the upper digestive tract [9,39]. The number of high-risk types of BPV found is an important factor and all types present in this group were found in the study samples, totaling 14 samples from animals infected with high-risk BPV, which shows the wide distribution of these viral types in Brazil. However, none of these high-risk BPV types have been found in cancer samples in this study. One possible explanation could be that the animals might have less access to bracken fern, which has a synergistic effect on the development of cancer in cattle [4,9,38].

Ten isolates were characterized as putative new types of BPV, phylogenetically closer to BPV3, BPV12, BPV24 and BPV26. Despite having a greater proximity to these types, the PNT showed relatively low identity with the reference BPV types; less than 80% with the exception of two isolates BPVUFSBR19 and BPVUFSBR20, which presented 87.8% and 86%, respectively. The data show that these 10 isolates probably belong to at least five different new types of BPV. The isolates BPVUFSBR20, BPVUFSBR30 and BPVUFSBR44 showed, respectively, 86%, 85% and 79% identity with the isolates found in Southern Brazil [40] and in China. This demonstrates that, to the best of our knowledge, these three isolates, together with the BPVUFSBR19 isolate, are BPV types that were found for the first time in this study. The other six isolates showed identity higher than 90% when compared with non-reference isolates possibly belonging to the same BPV type. Other studies have also reported the identification of putative new types of BPV [23,30,34,37,41], which evidences the great genetic diversity of BPV mostly in Brazil.

The identification and characterization of new types of BPV is of great importance for understanding the evolution of papillomaviruses, in addition to the clinical and epidemiological aspects related to diseases caused by these viruses [24]. In this epidemiological context, the results of this study show that some of the positive samples for BPV were from dairy cows subjected to artificial insemination with semen coming from another region of the country. Previous studies [35,42] have already reported the possibility of transmission of BPV by semen. This hypothesis may point to one of the possible ways of spreading the virus in the country, since the detection of BPV is not mandatory in semen marketed in Brazil [43,44].

It is important to highlight the number of BPV variants and subtypes found in the region, indicating that the virus has been undergoing many mutations in the L1 gene. To the best of the authors’ knowledge, BPV1, BPV2, BPV5, BPV8, BPV11, BPV13 and BPV26 presented mutations never reported previously. In addition, in this study it was observed that only four samples did not show mutations and out of the 38 that did, 22 samples had non-synonymous mutations, which were predicted to confer instability in the L1 protein structure of almost all isolates except one. Although the L1 gene is considered to be the most conserved gene for papillomaviruses, one study also reported a high genetic variability of the HPV L1 gene, showing that some regions of this gene, especially the regions related to loops in the L1 protein, are highly variable [33].

Studies with the evaluation of HPV neutralizing epitopes and genetic variability in these regions have demonstrated the possibility that mutations in certain L1 regions may have an effect on the host immune response to the virus [32,33]. By mapping these regions into the BPV L1 protein, it was possible to observe similar mutations in some samples of the present study, which may be associated with a possible change in the recognition of the host immune system, which may interfere with the viral infectivity. In the analyzed samples, 14 mutations occurred in the regions of hypervariable surface loops related to the neutralizing epitopes [32,33]. Out of these 14 mutations, seven were predicted to reduce the stability of the L1 protein, suggesting a possible interference in the infectivity of these BPV variants.

Two BPV4 variants were identified in fibropapillomatous skin lesions, which is not common. It is very likely that the genetic variability of BPVs can be associated with different morphological lesions. To validate this assertion, it would be necessary to study the expression of the early (E) proteins. Neoplastic lesions have been suggested to be associated with abortive infections in which the expression of the E proteins, mainly E5, has a pivotal role [9,45]. Therefore, further studies are needed to investigate the role of these BPV4 variants in fibropapillomatous lesions and assessing the genetic changes in their E proteins expression, as BPV4 is an epitheliotropic virus and is usually associated with tumors of the upper gastrointestinal tract.

In this study, a high genotype diversity of putative new types and intra-genotype variants of BPV in Brazil was identified, showing that BPV can be as genetically diverse as HPV and that further studies need to be carried out in order to identify this variability and characterize the genome of new BPV types, subtypes and variants. In this way, this study can serve as a basis to better understand the genetic diversity of BPV and to develop more efficient treatment and diagnosis strategies.

## 4. Materials and Methods 

This is a cross-sectional study whose samples were obtained on livestock farms in the state of Sergipe, Northeastern Brazil, both for meat and milk production. In total, 61 samples of papillomatous lesions were collected from animals of both sexes. The lesions were observed in several places on the animals’ body and were mainly fibropapilomas in the dorsal and cervical regions. The lesions were removed with the aid of a scalpel, transported on ice and stored at −20 °C. The study was approved by the Ethics Committee for Research on Farm Animals of the Federal University of Sergipe, under protocol number 05/14 (approved in 11/12/2014, extended in 31/07/2019).

The viral DNA was extracted using the Wizard Genomic DNA Purification Kit (Promega, Madison, WI, USA), following the protocols provided by the manufacturer. The samples were tested for the presence of BPV DNA by amplifying a fragment of the L1 gene by PCR, using degenerate primers FAP59/64 [46], which amplify a product with approximately 450 bp, using 10 min of initial denaturation at 94 °C, 40 cycles with three steps of one minute each, denaturation at 94 °C, annealing at 50 °C and extension at 72 °C in addition to a final 10 min extension at 72 °C. The amplified products were submitted to electrophoresis in 1.5% agarose gel, visualized and photographed in a UV transluminator. Known BPV samples, confirmed by sequencing, were used as positive controls. A PCR reaction mix containing ultrapure water instead of the DNA sample was used as a negative control.

After confirming the presence of viral DNA, the PCR product was purified using the Wizard SV Gel and PCR Clean-Up System kit (Promega) using the manufacturer’s protocol. Sequencing was then performed in duplicates using the ABI PRISM BigDye Terminator Cycle Sequencing v.3.1 kit (Applied Biosystems, Waltham, MA, USA) in a Sanger Sequencing 3500 Genetic Analyzer (Applied Biosystems, Waltham, MA, USA).

To ensure the quality of the sequencing and the assembly of the contigs, Pregap4 and Gap4 tools from the Staden package were used [47]. To guarantee the quality and formation of the contigs, sequencing was carried out twice, forward and reverse. Mean values of Phred equal to or greater than 30 were used as a quality cutoff for the sequences. In addition, regions at the beginning and at the end of the sequences with low similarity after a BLAST search were discarded to ensure the reliability of the DNA bases. The sequences were analyzed using Nucleotide BLAST [48] using MegaBLAST in order to compare with the reference BPV sequences deposited in the GenBank database (http://www.ncbi.nlm.nih.gov/genbank/).

The Muscle tool [49], incorporated into the MEGA-X software [50], was used to perform the multiple alignment of the sequences found in this study with those from the database. A phylogenetic tree was built using jModelTest [51] for model selection using the evolutionary model GTR + I + G that most fit the data and PhyML [52] for the reconstruction of the phylogenetic tree using the maximum likelihood method. Tree topology was optimized and the initial tree was defined by the neighbor-joining method. The best of the NNI and SPR methods were used for heuristic searches. In order to evaluate branch support, 1000 bootstrap replicates were used.

The Modeller program [53] was used with the standard parameters to build the three-dimensional models of the L1 protein, which was encoded by the L1 gene of all BPV types. This algorithm predicted the protein structure using a comparative method, allowing the evaluation of the structural effects of the mutations found in the BPV L1 proteins. The crystal structure of the BPV1 (PDB code: 3IYJ) and HPV16 (PDB code: 1DZL) L1 proteins, obtained from the Protein Data Bank (https://www.rcsb.org/), were used as templates. ModRefiner [54] and 3Drefine [55] were used with the standard parameters to refine the models through an energy minimization process. Procheck [56] was used to evaluate the stereochemical quality of the models through the analysis of the Ramachandran plot. Simulations were then performed using the Site Directed Mutator server [57] in order to evaluate the effect of each non-synonymous mutation on the L1 protein stability.

The sequences obtained in this study were deposited in GenBank and the respective accession numbers are described in Table S13.

## Figures and Tables

**Table 1:** Isolates identified as putative new types of BPV, their identity with the reference sequence, the closest reference type described and the GenBank sequences that showed greater identity with these samples.

Sample	Identity	Reference Type	Isolate with the Highest Identity	GenBank Accession Number
BPVUFSBR-19	87.8%	BPV26	BPV26: 87.8%	MG281846
BPVUFSBR-20	86.06%	BPV26	BR-UEL2: 86.62%	EU293538
BPVUFSBR-28	76.39%	BPV3	BR-UEL6: 95.92%	KP892554
BPVUFSBR-30	78.36%	BPV26	BR-UEL2: 85.67%	GQ471901
BPVUFSBR-31	77.25%	BPV3	BR-UEL6: 98.42%	KP892554
BPVUFSBR-34	77.81%	BPV12	06AC14: 93.88%	KP701433
BPVUFSBR-36	79.57%	BPV24	BR-UEL3: 99.78%	EU293539
BPVUFSBR-44	78.38%	BPV3	SW03: 79.21%	KF751802
BPVUFSBR-45	77.63%	BPV12	06AC14: 93.88%	EU293539
BPVUFSBR-49	74.13%	BPV3	BR-UEL6: 98.43%	KX924620

**Table 2:** Analysis of the effect of mutations on the structural stability of the BPV L1 protein based on the Site Directed Mutator server. The stability of the mutation is related to changes in the ΔΔG energy of the molecule with reduced stability for those mutations that have negative energy and increased stability for those that have positive energy. The data were clustered by BPV types.

BPV Type	Mutation	ΔΔG	Structure Stability
BPV1	I53N	−1.65	Reduced
	A55D	−0.02	Reduced
	A63K	−2.79	Reduced
	R105I	0.76	Increased
	L176P	0.58	Increased
BPV2	A134T	−0.93	Reduced
	L176P	−1.46	Reduced
	N178T	0.05	Increased
BPV11	K189R	−0.16	Reduced
BPV14	H220P	−1.98	Reduced
	I223K	−2.6	Reduced
BPV24	N132G	0.9	Increased
	D139E	0.53	Increased
BPV25	A168P	−1.37	Reduced
BPV26	A57G	0.57	Increased
	P85Q	−0.08	Reduced
	Y90N	−2.24	Reduced
	P92H	0.76	Increased
	S133G	0.59	Increased
	N144K	0.82	Increased
	C173G	−0.29	Reduced
	N190I	1.7	Increased

## Supplementary Materials

The following are available online at https://www.mdpi.com/2076-0817/9/9/748/s1, Table S1. Occurrence of co-infection with multiple BPV types in the animals, Table S2: Total number of variants and subtypes for each BPV type identified in this study, Table S3: Mutations found in the BPV1 L1 gene sequence. The dot (.) symbolizes that the site is the same as the reference sequence, Table S4: Mutations found in the BPV4 L1 gene sequences. The dot (.) symbolizes that the site is the same as the reference sequence, Table S5: Mutations found in the BPV5 L1 gene sequences, Table S6: Mutations found in the BPV11 L1 gene sequences. The dot (.) symbolizes that the site is the same as the reference sequence, Table S7: Mutations found in the BPV13 L1 gene sequences. The dot (.) symbolizes that the site is the same as the reference sequence, Table S8: Mutations found in the BPV14 L1 gene sequences. The dot (.) symbolizes that the site is the same as the reference sequence, Table S9: Mutations found in the BPV24 L1 gene sequences. The dot (.) symbolizes that the site is the same as the reference sequence, Table S10: Mutations found in the BPV25 L1 gene sequences. The dot (.) symbolizes that that site is the same as the reference sequence. The hyphen (-) symbolizes a non-sequenced site, Table S11: Mutations found in the BPV2 L1 gene sequences. The dot (.) symbolizes that that site is the same as the reference sequence. The hyphen (-) symbolizes a non-sequenced site, Table S12: Mutations found in the BPV26 L1 gene sequences. The dot (.) symbolizes that that site is the same as the reference sequence. The hyphen (-) symbolizes a non-sequenced site, Table S13: GenBank accession number of the sequences obtained in this study.
