# Supplementary material for: High Genotypic Diversity, Putative New Types and Intra-Genotype Variants of Bovine Papillomavirus in Northeast Brazil
Source: Pathogens. 2020 Sep 15;9(9):748. doi: 10.3390/pathogens9090748 (PMC7558504; doi:10.3390/pathogens9090748)
Supplement: Supplementary file 1 [file pathogens-09-00748-s001.zip › pathogens-909627supp.pdf]

Table S1. Total number of variants and subtypes for each BPV type identified in this study.

BPV TYPE	VARIANTS	SUBTYPES	TOTAL
**BPV1**	1	0	1
**BPV2**	5	2	7
**BPV4**	1	0	1
**BPV5**	0	2	2
**BPV11**	4	0	4
**BPV13**	3	0	3
**BPV14**	1	0	1
**BPV24**	1	0	1
**BPV25**	8	0	8
**BPV26**	6	4	10

Table S2. Mutations found in the BPV1 L1 gene sequence. The dot (.) symbolizes that the site is the same as the reference sequence.

DNA	158	159	164	187	188	314	330	527
**BPV1REF**	T	C	C	G	C	G	A	T
**BPVUFSBR-04**	A	T	A	A	A	T	G	C
**AMINO ACID**	**53**	**53**	**55**	**63**	**63**	**105**	**110**	**176**
**BPV1REF**	I	I	A	A	A	R	G	L
**BPVUFSBR-04**	N	N	D	K	K	I	.	P

Table S3. Mutations found in the BPV4 L1 gene sequences. The dot (.) symbolizes that the site is the same as the reference sequence.

DNA	399	408
**BPV4REF**	T	C
**BPVUFSBR-01**	G	T
**BPVUFSBR-03**	.	.

Table S4. Mutations found in the BPV5 L1 gene sequences.

DNA	147	156	174	349	351	354
**BPV5REF**	C	T	A	T	G	T
**BPVUFSBR-32**	T	C	T	C	C	C
**BPVUFSBR-33**	T	C	T	C	C	C

Table S5. Mutations found in the BPV11 L1 gene sequences. The dot (.) symbolizes that the site is the same as the reference sequence.

**DNA**	**327**	**566**	**588**
**BPV11REF**	A	A	T
**BPVUFSBR-13**	T	G	C
**BPVUFSBR-16**	T	.	C
**BPVUFSBR-17**	T	.	C
**BPVUFSBR-18**	T	.	C
**AMINO ACID**	**109**	**189**	**196**
**BPV11REF**	G	K	G
**BPVUFSBR-13**	.	R	.
**BPVUFSBR-16**	.	.	.
**BPVUFSBR-17**	.	.	.
**BPVUFSBR-18**	.	.	.

Table S6. Mutations found in the BPV13 L1 gene sequences. The dot (.) symbolizes that the site is the same as the reference sequence.

DNA	144	525	582
**BPV13REF**	C	G	A
**BPVUFSBR-14**	T	A	C
**BPVUFSBR-15**	T	A	C
**BPVUFSBR-29**	T	.	C

Table S7. Mutations found in the BPV14 L1 gene sequences. The dot (.) symbolizes that the site is the same as the reference sequence.

**DNA**	**659**	**668**	**681**
**BPV14REF**	A	T	T
**BPVUFSBR-12**	C	A	C
**AMINO ACID**	**220**	**223**	**227**
**BPV14REF**	H	I	D
**BPVUFSBR-12**	P	K	.

Table S8. Mutations found in the BPV24 L1 gene sequences. The dot (.) symbolizes that the site is the same as the reference sequence.

DNA	339	394	395	417
**BPV24REF**	T	A	A	T
**BPVUFSBR-35**	A	G	G	A
**AMINO ACID**	**113**	**132**	**132**	**139**
**BPV24REF**	G	N	N	D
**BPVUFSBR-35**	.	G	G	E

Table S9. Mutations found in the BPV25 L1 gene sequences. The dot (.) symbolizes that that site is the same as the reference sequence. The hyphen (-) symbolizes a non-sequenced site.

DNA	135	502	579
BPV25REF	A	G	A
BPVUFSBR-40	G	C	C
BPVUFSBR-41	G	C	C
BPVUFSBR-42	G	C	C
BPVUFSBR-43	G	C	C
BPVUFSBR-46	G	C	C
BPVUFSBR-50	G	C	C
BPVUFSBR-51	G	C	C
BPVUFSBR-52	G	-	-
**AMINO ACID**	**45**	**168**	**193**
BPV25REF	V	A	G
BPVUFSBR-40	.	P	.
BPVUFSBR-41	.	P	.
BPVUFSBR-42	.	P	.
BPVUFSBR-43	.	P	.
BPVUFSBR-46	.	P	.
BPVUFSBR-50	.	P	.
BPVUFSBR-51	.	P	.
BPVUFSBR-52	.	-	-

Table S10. Mutations found in the BPV2 L1 gene sequences. The dot (.) symbolizes that that site is the same as the reference sequence. The hyphen (-) symbolizes a non-sequenced site.

**DNA**	**144**	**225**	**285**	**294**	**306**	**309**	**318**	**354**	**364**	**400**	**507**	**527**	**533**
**BPV2REF**	C	T	T	A	A	A	C	T	C	G	T	T	A
**BPVUFSBR-02**	-	-	G	C	G	G	.	A	.	.	-	-	-
**BPVUFSBR-05**	-	.	G	C	.	G	.	.	T	.	-	-	-
**BPVUFSBR-06**	-	-	G	.	.	G	.	.	.	.	-	-	-
**BPVUFSBR-07**	-	.	G	.	.	G	.	.	.	.	-	-	-
**BPVUFSBR-08**	-	.	G	.	.	G	.	.	.	.	-	-	-
**BPVUFSBR-10**	T	C	G	C	.	G	A	.	.	A	C	C	C
**BPVUFSBR-11**	T	.	G	C	.	G	.	.	.	.	.	-	-
**AMINO ACID**	**48**	**75**	**95**	**98**	**102**	**103**	**106**	**118**	**122**	**134**	**169**	**176**	**178**
**BPV2REF**	Y	D	V	V	Q	V	G	T	L	A	R	L	N
**BPVUFSBR-02**	-	-	.	.	.	.	.	.	.	.	-	-	-
**BPVUFSBR-05**	-	.	.	.	.	.	.	.	.	.	-	-	-
**BPVUFSBR-06**	-	-	.	.	.	.	.	.	.	.	-	-	-
**BPVUFSBR-07**	-	.	.	.	.	.	.	.	.	.	-	-	-
**BPVUFSBR-08**	-	.	.	.	.	.	.	.	.	.	-	-	-
**BPVUFSBR-10**	.	.	.	.	.	.	.	.	.	T	.	P	T
**BPVUFSBR-11**	.	.	.	.	.	.	.	.	.	.	.	-	-

Table S11. Mutations found in the BPV26 L1 gene sequences. The dot (.) symbolizes that that site is the same as the reference sequence. The hyphen (-) symbolizes a non-sequenced site.

**DNA**	**147**	**156**	**157**	**170**	**177**	**192**	**201**	**217**	**219**	**234**	**237**
**BPV26REF**	A	T	T	C	A	T	C	A	G	C	T
**BPVUFSBR-21**	.	.	.	G	.	.	.	.	.	.	.
**BPVUFSBR-22**	.	.	.	G	.	.	.	.	.	.	.
**BPVUFSBR-23**	.	.	.	G	.	.	.	.	.	.	.
**BPVUFSBR-24**	.	.	.	G	.	.	.	.	.	.	.
**BPVUFSBR-25**	.	.	.	G	.	.	.	.	.	.	.
**BPVUFSBR-26**	.	.	.	G	.	.	.	.	.	.	.
**BPVUFSBR-27**	G	.	.	G	.	C	T	.	.	T	.
**BPVUFSBR-37**	.	C	C	G	T	.	T	C	A	.	A
**BPVUFSBR-38**	.	C	C	G	T	.	T	C	A	.	A
**BPVUFSBR-39**	.	C	C	G	T	.	T	C	A	.	A
**AMINO ACID**	**49**	**52**	**53**	**57**	**59**	**64**	**67**	**73**	**73**	**78**	**79**
**BPV26REF**	P	D	L	A	T	V	S	R	R	D	P
**BPVUFSBR-21**	-	.	.	G	.	.	.	.	.	.	.
**BPVUFSBR-22**	.	.	.	G	.	.	.	.	.	.	.
**BPVUFSBR-23**	.	.	.	G	.	.	.	.	.	.	.
**BPVUFSBR-24**	.	.	.	G	.	.	.	.	.	.	.
**BPVUFSBR-25**	.	.	.	G	.	.	.	.	.	.	.
**BPVUFSBR-26**	.	.	.	G	.	.	.	.	.	.	.
**BPVUFSBR-27**	.	.	.	G	.	.	.	.	.	.	.
**BPVUFSBR-37**	.	.	.	G	.	.	.	.	.	.	.
**BPVUFSBR-38**	.	.	.	G	.	.	.	.	.	.	.
**BPVUFSBR-39**	.	.	.	G	.	.	.	.	.	.	.

Table S11. (Continuation) Mutations found in the BPV26 L1 gene sequences. The dot (.) symbolizes that that site is the same as the reference sequence. The hyphen (-) symbolizes a non-sequenced site.

DNA	254	268	275	279	285	286	288	306	312	318	324
BPV26REF	C	T	C	C	A	C	G	G	C	T	G
BPVUFSBR-21	A	.	.	.	.	.	.	.	.	A	.
BPVUFSBR-22	A	.	.	.	.	.	.	.	.	A	.
BPVUFSBR-23	A	.	.	.	.	.	.	.	.	A	.
BPVUFSBR-24	A	.	.	.	.	.	.	.	.	A	.
BPVUFSBR-25	A	.	.	.	.	.	.	.	.	A	.
BPVUFSBR-26	A	.	.	.	.	.	.	.	.	A	.
BPVUFSBR-27	A	A	A	.	.	.	.	.	.	A	.
BPVUFSBR-37	A	.	.	T	G	A	A	A	A	A	A
BPVUFSBR-38	A	.	.	T	G	A	A	A	A	A	A
BPVUFSBR-39	A	.	.	T	G	A	A	A	A	A	A
**AMINO ACID**	**85**	**90**	**92**	**93**	**95**	**96**	**96**	**102**	**104**	**106**	**108**
BPV26REF	P	Y	P	D	Q	R	R	R	I	I	R
BPVUFSBR-21	Q	.	.	.	.	.	.	.	.	.	.
BPVUFSBR-22	Q	.	.	.	.	.	.	.	.	.	.
BPVUFSBR-23	Q	.	.	.	.	.	.	.	.	.	.
BPVUFSBR-24	Q	.	.	.	.	.	.	.	.	.	.
BPVUFSBR-25	Q	.	.	.	.	.	.	.	.	.	.
BPVUFSBR-26	Q	.	.	.	.	.	.	.	.	.	.
BPVUFSBR-27	Q	N	H	.	.	.	.	.	.	.	.
BPVUFSBR-37	Q	.	.	.	.	.	.	.	.	.	.
BPVUFSBR-38	Q	.	.	.	.	.	.	.	.	.	.
BPVUFSBR-39	Q	.	.	.	.	.	.	.	.	.	.

Table S11. (Continuation) Mutations found in the BPV26 L1 gene sequences. The dot (.) symbolizes that that site is the same as the reference sequence. The hyphen (-) symbolizes a non-sequenced site.

**DNA**	**327**	**330**	**336**	**345**	**351**	**378**	**390**	**397**	**432**	**441**	**447**	**462**
**BPV26REF**	C	G	G	A	A	A	T	A	T	A	C	A
**BPVUFSBR-21**	.	.	.	.	C	.	.	G	.	.	G	.
**BPVUFSBR-22**	.	.	.	.	C	.	.	G	.	.	G	.
**BPVUFSBR-23**	.	.	.	.	C	.	.	G	.	.	G	.
**BPVUFSBR-24**	.	.	.	.	C	.	.	G	.	.	G	.
**BPVUFSBR-25**	.	.	.	.	C	.	.	G	.	.	G	.
**BPVUFSBR-26**	.	.	.	.	C	.	.	G	.	.	G	.
**BPVUFSBR-27**	.	.	.	.	C	G	.	G	A	.	.	T
**BPVUFSBR-37**	A	A	A	T	.	G	C	.	.	G	.	T
**BPVUFSBR-38**	A	A	A	T	.	G	C	.	.	G	.	T
**BPVUFSBR-39**	A	A	A	T	.	G	C	.	.	G	.	T
**AMINO ACID**	**109**	**110**	**112**	**115**	**117**	**126**	**130**	**133**	**144**	**147**	**149**	**154**
**BPV26REF**	G	Q	L	G	T	K	N	S	N	V	P	L
**BPVUFSBR-21**	.	.	.	.	.	.	.	G	.	.	.	.
**BPVUFSBR-22**	.	.	.	.	.	.	.	G	.	.	.	.
**BPVUFSBR-23**	.	.	.	.	.	.	.	G	.	.	.	.
**BPVUFSBR-24**	.	.	.	.	.	.	.	G	.	.	.	.
**BPVUFSBR-25**	.	.	.	.	.	.	.	G	.	.	.	.
**BPVUFSBR-26**	.	.	.	.	.	.	.	G	.	.	.	.
**BPVUFSBR-27**	.	.	.	.	.	.	.	G	K	.	.	.
**BPVUFSBR-37**	.	.	.	.	.	.	.	.	.	.	.	.
**BPVUFSBR-38**	.	.	.	.	.	.	.	.	.	.	.	.
**BPVUFSBR-39**	.	.	.	.	.	.	.	.	.	.	.	.

Table S11. (Continuation) Mutations found in the BPV26 L1 gene sequences. The dot (.) symbolizes that that site is the same as the reference sequence. The hyphen (-) symbolizes a non-sequenced site.

**DNA**	**474**	**480**	**486**	**492**	**498**	**517**	**546**	**562**	**564**	**569**	**576**	**579**
**BPV26REF**	T	G	C	A	T	T	C	T	A	A	A	A
**BPVUFSBR-21**	.	.	.	G	.	.	.	.	.	.	.	.
**BPVUFSBR-22**	.	.	.	G	.	.	.	.	.	.	.	.
**BPVUFSBR-23**	.	.	.	G	.	.	.	.	.	.	.	.
**BPVUFSBR-24**	.	.	.	G	.	.	.	.	.	.	.	.
**BPVUFSBR-25**	.	.	.	G	.	.	.	.	.	.	.	.
**BPVUFSBR-26**	.	.	.	G	.	.	.	.	.	.	.	.
**BPVUFSBR-27**	.	.	T	.	.	G	T	.	.	T	.	.
**BPVUFSBR-37**	C	A	T	.	C	.	T	C	G	.	G	T
**BPVUFSBR-38**	C	A	T	.	C	.	T	C	G	.	G	T
**BPVUFSBR-39**	C	A	T	.	C	.	T	C	G	.	G	T
**AMINO ACID**	**158**	**160**	**162**	**164**	**166**	**173**	**182**	**188**	**188**	**190**	**192**	**193**
**BPV26REF**	G	V	C	G	H	C	D	L	L	N	K	I
**BPVUFSBR-21**	.	.	.	.	.	.	.	.	.	.	.	.
**BPVUFSBR-22**	.	.	.	.	.	.	.	.	.	.	.	.
**BPVUFSBR-23**	.	.	.	.	.	.	.	.	.	.	.	.
**BPVUFSBR-24**	.	.	.	.	.	.	.	.	.	.	.	.
**BPVUFSBR-25**	.	.	.	.	.	.	.	.	.	.	.	.
**BPVUFSBR-26**	.	.	.	.	.	.	.	.	.	.	.	.
**BPVUFSBR-27**	.	.	.	.	.	G	.	.	.	I	.	.
**BPVUFSBR-37**	.	.	.	.	.	.	.	.	.	.	.	.
**BPVUFSBR-38**	.	.	.	.	.	.	.	.	.	.	.	.
**BPVUFSBR-39**	.	.	.	.	.	.	.	.	.	.	.	.

Table S12. GenBank accession number of the sequences obtained in this study.

BPV ISOLATE	GENBANK ACCESSION NUMBER
BPVUFSBR-01	MT674570
BPVUFSBR-02	MT674571
BPVUFSBR-03	MT674572
BPVUFSBR-04	MT674573
BPVUFSBR-05	MT674574
BPVUFSBR-06	MT674575
BPVUFSBR-07	MT674576
BPVUFSBR-08	MT674577
BPVUFSBR-09	MT674578
BPVUFSBR-10	MT674579
BPVUFSBR-11	MT674580
BPVUFSBR-12	MT674581
BPVUFSBR-13	MT674582
BPVUFSBR-14	MT674583
BPVUFSBR-15	MT674584
BPVUFSBR-16	MT674585
BPVUFSBR-17	MT674586
BPVUFSBR-18	MT674587
BPVUFSBR-19	MT674588
BPVUFSBR-20	MT674589
BPVUFSBR-21	MT674590
BPVUFSBR-22	MT674591
BPVUFSBR-23	MT674592
BPVUFSBR-24	MT674593
BPVUFSBR-25	MT674594
BPVUFSBR-26	MT674595
BPVUFSBR-27	MT674596
BPVUFSBR-28	MT674597
BPVUFSBR-29	MT674598
BPVUFSBR-30	MT674599
BPVUFSBR-31	MT674600
BPVUFSBR-32	MT674601
BPVUFSBR-33	MT674602
BPVUFSBR-34	MT674603
BPVUFSBR-35	MT674604
BPVUFSBR-36	MT674605
BPVUFSBR-37	MT674606
BPVUFSBR-38	MT674607
BPVUFSBR-39	MT674608
BPVUFSBR-40	MT674609
BPVUFSBR-41	MT674610
BPVUFSBR-42	MT674611
BPVUFSBR-43	MT674612
BPVUFSBR-44	MT674613
BPVUFSBR-45	MT674614
BPVUFSBR-46	MT674615
BPVUFSBR-47	MT674616
BPVUFSBR-48	MT674617
BPVUFSBR-49	MT674618
BPVUFSBR-50	MT674619
BPVUFSBR-51	MT674620
BPVUFSBR-52	MT674621
